# Morphology controlled synthesis of 2-D Ni–Ni_3_S_2_ and Ni_3_S_2_ nanostructures on Ni foam towards oxygen evolution reaction

**DOI:** 10.1186/s40580-017-0101-6

**Published:** 2017-03-28

**Authors:** Nitin Kaduba Chaudhari, Aram Oh, Young Jin Sa, Haneul Jin, Hionsuck Baik, Sang Gu Kim, Suk Joong Lee, Sang Hoon Joo, Kwangyeol Lee

**Affiliations:** 1 Department of Chemistry and Research Institute for Natural Sciences, Korea University, Seoul, 02841 Republic of Korea; 2 Center for Molecular Spectroscopy and Dynamics, Institute for Basic Science (IBS), Seoul, 02841 Republic of Korea; 3 Department of Chemistry, Ulsan National Institute of Science and Technology (UNIST), Ulsan, 44919 Republic of Korea; 4 Korea Basic Science Institute (KBSI), Seoul, 02841 Republic of Korea

**Keywords:** Nickel sulphide, OER, Tafel slope, Nickel foam, Nickel nanosheets

## Abstract

Catalysts for oxygen evolution reactions (OER) are at the heart of key renewable energy technologies, and development of non-precious metal catalysts with high activity and stability remain a great challenge in this field. Among various material candidates, metal sulfides are receiving increasing attention. While morphology-dependent catalytic performances are well established in noble metal-based catalysts, relatively little is known for the morphology‒catalytic performance relationship in metal sulfide catalysts. In this study, uniform spider web-like Ni nanosheets–Ni_3_S_2_ and honeycomb-like Ni_3_S_2_ structures are deposited on nickel foam (Ni_3_S_2_/NF) by a facile one-step hydrothermal synthetic route. When used as an oxygen evolution electrode, the spider web-like Ni–Ni_3_S_2_/NF with the large exposed surface area shown excellent catalytic activity and stability with an overpotential of ~310 mV to achieve at 10 mA/cm^2^ and a Tafel slope of 63 mV/dec in alkaline media, which is superior to the honeycomb-like structure without Ni nanosheet. The low Tafel slope of the spider web-like Ni–Ni_3_S_2_/NF represents one of the best OER kinetics among nickel sulfide-based OER catalysts. The results point to the fact that performance of the metal sulfide electrocatalysts might be fine-tuned and optimized with morphological controls.

**Electronic supplementary material:**

The online version of this article (doi:10.1186/s40580-017-0101-6) contains supplementary material, which is available to authorized users.

## Background

Rapid depletion of fossil fuel and growing concern over global warming have motivated the ever-increasing interest in a clean energy carrier, hydrogen. Water electrolysis has been considered as a promising, sustainable route to hydrogen production. In water electrolysers, the anodic oxygen evolution reaction (OER) involves energetically demanding proton-coupled four electron transfer, and hence the development of highly active and stable electrocatalysts for the oxygen evolution reaction (OER) has been a major challenge. To date, the best OER electrocatalysts in terms of activity and stability have been RuO_2_ and IrO_2_ [1–6], which are not immune from the high production cost. Therefore, one of the foremost challenges is to develop highly active, yet inexpensive OER catalysts. Various transition metal oxide based materials have been investigated for such purposes [7–15]. However, the overpotentials of these materials are, in general, higher than that of RuO_2_ [16]. As a result, there remains a great need to identify and discover new compositions of electrocatalysts with highly efficient and low-cost for OER, which can compete with precious metal-based catalysts.

Due to the better stability and excellent corrosion resistance of Ni-based materials in the alkaline media, researchers are interested in using nickel alloys or their composites as electrode materials for OER and HER [17–19] Additionally, over the last few years, considerable research effort has been devoted to the design and synthesis of metal sulfides as water splitting electrode catalyst, with the aim of attaining oxygen evolution at low overpotential [20–26]. Ni sulfide (Ni_3_S_2_), in particular, is of a great interest due to its high conductivity, low cost, facile preparation, and high catalytic activity [27–30]. Importantly, Ni_3_S_2_ possesses higher stability in the presence of oxygen as compared to other Ni–S phases; sulfur atoms in other Ni–S phases can be quickly replaced by oxygen atoms to form Ni oxide [31]. However, the catalytic activity and stability of Ni_3_S_2_ toward OER are still less pronounced than those of noble metal catalysts [32–36].

Furthermore, the electrocatalytic performance of nanocatalysts is strongly dependent on the structural features, and designing nanostructures that can expose a high density of active sites is of critical importance [24, 33–39]. Therefore, control of reaction sites with uniquely exposed active surface planes in the electrocatalysts should be an effective strategy for boosting the electrochemical performances. Advantageously, the thin nanosheets can serve as an ideal platform to achieve the above mentioned goal. The two dimensional nanosheets could exclusively expose the specific crystal planes with active sites. Based on the above considerations, the synergistic manipulation of exposed surface planes of nanosheets and the nanostructured metal sulfide material would be an efficient strategy to pursue efficient electrocatalysts under the alkaline solutions. Nickel would be a competitive candidate because it offers corrosion resistance and high OER activity making it an excellent candidate material to be deposited on the metal sulfide, which could ensure fast charge migration during the electrocatalysts pathway [40]. However, the preparation and deposition of the porous nickel film and their compositions is complicated and difficult [41]. Furthermore, nickel films as a electrocatalyst have been rarely explored towards the OER [42]. As a result, it is still greatly challengeable to explore and develop facile yet effective approach to fabricate highly exposed porous nickel nanosheets.

Herein, we report the controllable synthesis of two different nickel sulfide based nanostructures on Ni foam, spider web-like Ni–Ni_3_S_2_ and honeycomb-like Ni_3_S_2_ denoted as SW Ni–Ni_3_S_2_/NF and HC–Ni_3_S_2_/NF, respectively. The use of lower precursor concentration could achieve a uniform deposition of spider web-like Ni-nanosheets on the Ni_3_S_2_ while an increase in precursor concentration could lead to simply honey-comb like Ni_3_S_2_ nanostructure. Interestingly, detailed investigations reveal that the formation of Ni-nanosheets is accomplished by initial formation of densely packed pillared Ni_3_S_2_ structure grown on NF and subsequent deposition of spider web-like Ni nanosheets. On the other hand, HC–Ni_3_S_2_/NF with uniform honeycomb-like nanostructure is obtained on NF at a high precursor concentration. The key strategy in this work is to develop a synergetic effect between morphologically controlled Ni nanosheets and Ni_3_S_2_. Current density of 10 mA/cm^2^ at overpotential of 310 mV and Tafel slope of 63 mV/dec have been recorded with SW Ni–Ni_3_S_2_/NF, which is, to the best of our knowledge, the lowest Tafel slope recorded for the Ni_3_S_2_ based electrocatalysts for OER, while Tafel slope value of 110 mV/dec was found for HC–Ni_3_S_2_/NF.

## Experimental section

### Synthesis of SW Ni–Ni_3_S_2_/NF and HC–Ni_3_S_2_/NF nanostructures

Spider web-like Ni–Ni_3_S_2_ on NF (SW Ni–Ni_3_S_2_/NF) were prepared via a one-step template-free hydrothermal route. Prior to the synthesis, the NF substrates (length × diameter × thickness = 2.5 × 0.5 × 0.1 cm, 100 PPI, WELCOS Co., Ltd, South Korea) were cleaned by sonication in water, acetone, and then ethanol for 20 min each, and dried in an oven. The cleaned piece of NF substrate was then placed standing against the wall of a Teflon-lined autoclave (45 mL) containing 0.35 mmol NiCl_2_·6H_2_O, 0.25 mmol thiourea (CH_4_N_2_S), 2 mL of ethylene glycol and 8 mL DI water. Then, the autoclave was sealed and gradually heated and maintained at 160 °C for 14 h. After the hydrothermal reaction the slightly greenish coloured Ni-foam containing Ni–Ni_3_S_2_ were taken out and washed repeatedly using DI water and ethanol in order to remove the residual debris, and dried in an air oven at 80 °C overnight. Honeycomb-like Ni_3_S_2_ on NF (HC–Ni_3_S_2_/NF) nanostructures were synthesized under the same conditions mentioned above, except that 1.4 mmol nickel chloride and 1.0 mmol thiourea were used.

### Material characterizations

The samples were examined by X-ray diffraction (XRD) analysis with a Rigaku Ultima III diffractometer system using a graphite-monochromatized Cu-Kα radiation at 40 kV and 40 mA. The field emission scanning electron microscopy (FE-SEM) was used to examine morphology of the samples using a JEOL JSM-7001F machine. Transmission electron microscopy (TEM) and high-resolution TEM (HR-TEM) images were taken with a TECNAI G2 20 S-Twin operated at 200 kV and TECNAI G2 F30 operated at 300 kV, respectively.

### Electrochemical measurements

The OER activity of the catalysts were measured using an electrochemical workstation (IviumStat, Ivium Technologies) in a conventional three-electrode configuration with 1 M KOH electrolyte (99.99%, Aldrich) at a room temperature. The SW Ni–Ni_3_S_2_/NF and HC–Ni_3_S_2_/NF [1 × 1 cm; active area = 2.0 cm^2^ (both sides)] was used as the working electrode. An Hg/HgO (CHI152, with 1 M KOH filling solution) electrode and a graphite rod were used as the reference and counter electrode, respectively. The potentials were converted to the reversible hydrogen electrode (RHE) using the following equation:

$${\text{E}}_{{({\text{RHE}})}} = {\text{E}}_{{({\text{Hg}}/{\text{HgO}})}} + 0.0 5 9 \times {\text{pH}} + 0.0 9 8\,{\text{V}} = {\text{E}}_{{({\text{Hg}}/{\text{HgO}})}} + 0. 9 2 4\,{\text{V}}.$$

To obtain the series resistance for *iR*-compensation, electrochemical impedance spectroscopy was conducted at 1.4 V (vs RHE) from 100 kHz to 1 Hz. *x*-intercept at high frequency region was determined as the series resistance. Cyclic voltammetry (CV) was carried out from 1.1 to 1.7 V (vs RHE) at scan rate of 1 mV/s. Post *iR*-correction was then performed. Stability of the catalysts was tested by chronopotentiometry at 1.7 V (vs RHE without *iR*-compensation) in 1 M KOH for ~24 h. For comparison, OER activity of a bare NF was also measured in the same manner as described above. The mass of Ni–Ni_3_S_2_ and Ni_3_S_2_ grown on the Ni foam was calculated as following: the weight increment (x mg) of Ni foam can be directly weighted after the synthesis of Ni–Ni_3_S_2_ and Ni_3_S_2_ on Ni foam. M_Ni–Ni3S2_ = x mg × (M_Ni–Ni3S2_/2M_S_) = x mg × (267/64) = 4.17x mg and m_Ni3S2_ = x mg × (M_Ni3S2_/2M_S_) = x mg × (240/64) = 3.75x mg, where M is the molecular weight or atomic weight. For SW Ni–Ni_3_S_2_ and HC–Ni_3_S_2_ the loading mass was about 3.3 and 16 mg/cm^2^, respectively.

## Results and discussion

**Fig. 1:**
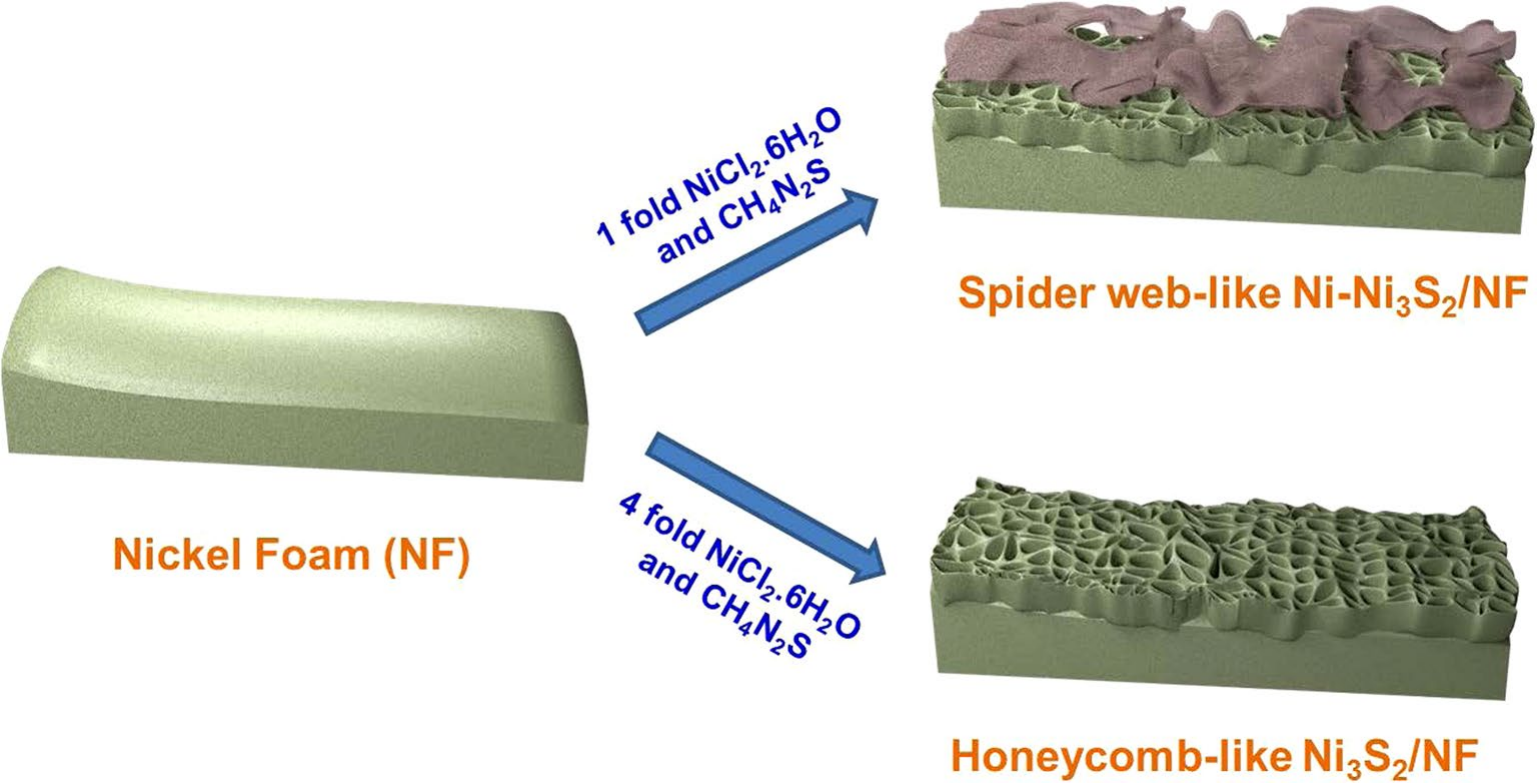
Schematic representation of the preparation of SW Ni–Ni_3_S_2_/NF and HC–Ni_3_S_2_/NF nanostructures

**Fig. 2:**
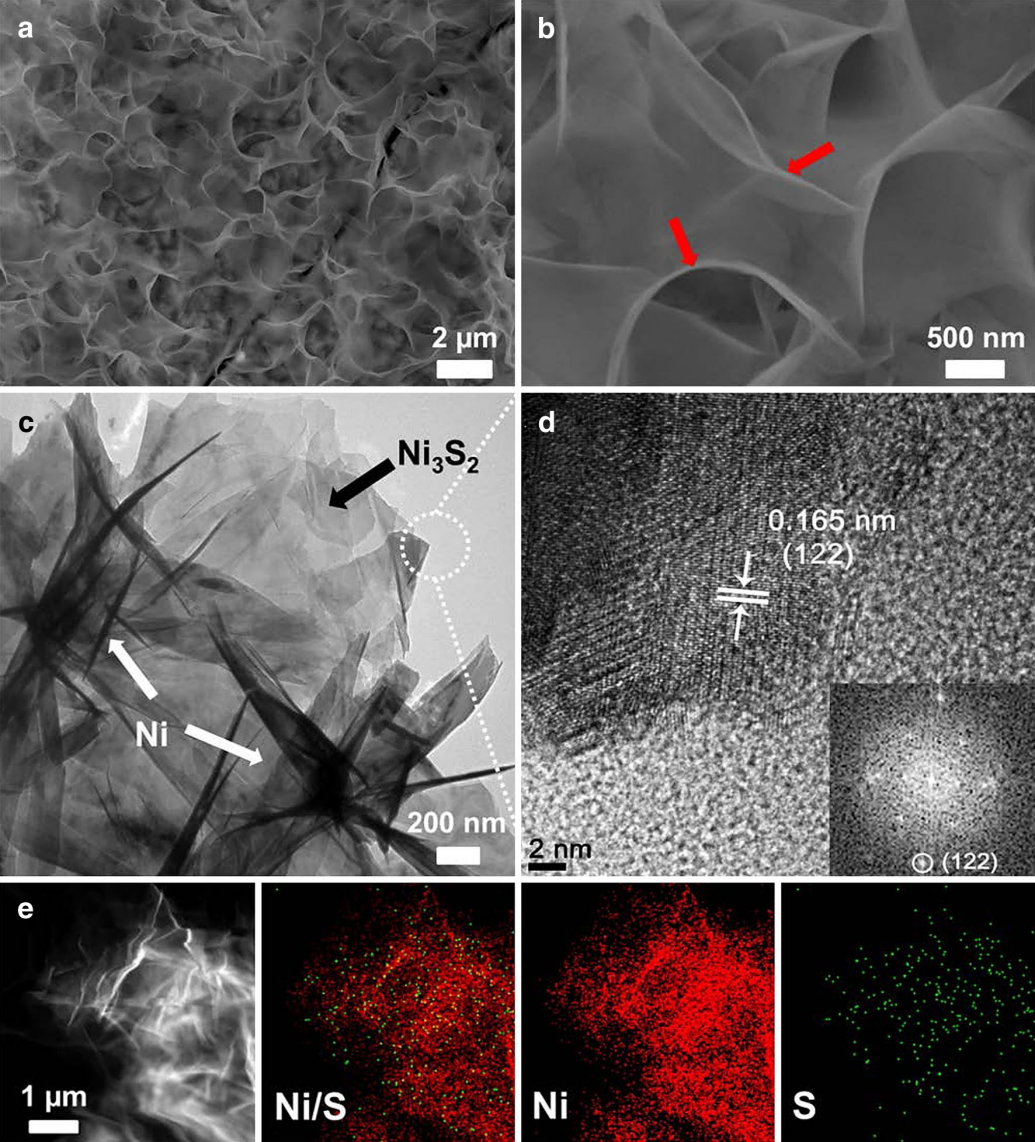
**a**, **b** FE-SEM images of SW Ni–Ni_3_S_2_/NF, **c** TEM image, **d** HR-TEM image, and **e** corresponding EDS elemental mapping of nickel nanosheets scrapped down from the Ni foam. The *arrows* in **b** show the* upright edges* and existence of spider on web-like Ni nanosheets

In a typical synthesis of spider web-like Ni–Ni_3_S_2_/NF nanostructures, NF was dipped into the aqueous solution of Ni chloride and thiourea in Teflon autoclave and heated at 160 °C for 14 h. After the hydrothermal reaction, the slightly greenish coloured Ni–Ni_3_S_2_/NF was removed, washed repeatedly using DI water and ethanol, and dried in an oven at 80 °C. Nanostructure of honeycomb like-Ni_3_S_2_/NF was prepared by increasing the amount of precursors (See “Experimental details” for more information). Figure 1 shows a schematic illustration of the preparation of spider web-like Ni–Ni_3_S_2_ and honeycomb-like Ni_3_S_2_ nanostructures on Ni foam. The morphology of these nanostructures was characterized by FE-SEM. At lower magnification, the NF surface is completely covered with Ni nanosheets (Additional file 1: Figure S1a and b). However, the magnified images (Fig. 2a, b) of as-obtained material indicate that thin Ni-nanosheets are interconnected with each other, forming a highly porous spider web on ground-like nanostructure. Thus, 2D architectural Ni-nanosheet surface, horizontally covering total Ni_3_S_2_ and NF surface, could be entirely exposed and highly accessible by the electrolyte when used as a catalyst for OER. TEM and HR-TEM measurements were carried out for as-prepared Ni–Ni_3_S_2_, which was scratched out from NF. The TEM image of Ni-nanosheets (Fig. 2c) is consistent with the result of FE-SEM analysis, showing multiple crumpled Ni-nanosheets (marked with white arrows) and pillared Ni_3_S_2_ nanostructure. The Ni_3_S_2_ nanostructures are highly crystalline as shown in Fig. 2d, and the lattice plane spacing of 0.16 nm can be indexed to the (122) plane of Ni_3_S_2_. For direct visualization to confirm the formation of Ni-nanosheets high-angle annular dark field (HAADF) imaging and energy dispersive X-ray spectroscopy (EDS) was carried out as shown in Fig. 2e. The scrambled nanosheets structure shows the main existence of Ni (more than 99%), traces of S (less than 1%) was also observed. The S element came from the pillared Ni_3_S_2_ structure present in the powder samples.

**Fig. 3:**
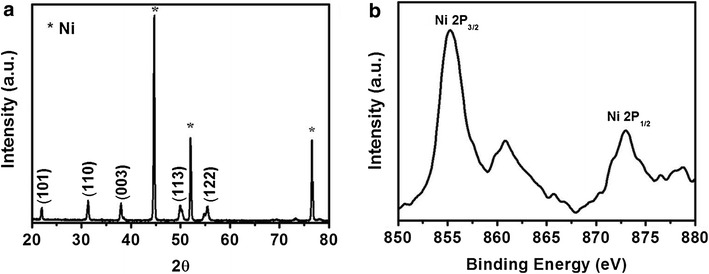
**a** X-Ray diffraction and **b** XPS spectra of the SW Ni–Ni_3_S_2_/NF

X-ray powder diffraction (XRD) pattern of SW Ni–Ni_3_S_2_/NF is presented in Fig. 3a. Except the peaks marked with asterisks at 2*θ* = 44.5°, 51.8°, and 76.4° (reflections of NF, JCPDS card No. 03-1051), all the peaks match well with diffractions of Ni_3_S_2_ (JCPDS card no. 44-1418). Due to the presence of NF substrate the Ni-nanosheets peaks could not be separated. However, strong and sharp peaks of the Ni_3_S_2_ suggest that the pillared structures are highly crystalline. No diffraction peaks other than Ni (originated from Ni-nanosheets and Ni-foam) and Ni_3_S_2_ are observed, suggesting the high purity of the as-obtained SW Ni–Ni_2_S_3_/NF material. In order to identify the oxidation states of Ni, X-ray photoelectron spectroscopy (XPS) study was also performed for SW Ni–Ni_3_S_2_/NF. As depicted in Fig. 3b, two strong major peaks at 855.3 and 872.9 eV in the XPS spectrum are assigned to Ni 2p_3/2_ and Ni 2p_1/2_, respectively [34] and the energy difference between Ni 2p_3/2_ (855.3 eV) and Ni 2p_1/2_ (872.9 eV) is observed to be 17.6 eV, suggesting the coexistence of Ni^2+^ and Ni^3+^ [43]. The XPS results are consistent with the presence of Ni_3_S_2_.

To understand the growth mechanism behind the successful synthesis of spider web-like Ni–Ni_3_S_2_ nanostructure, the products obtained from different stages of the reaction has been characterized (Additional file 1: Figure S2). The SEM analysis reveals that at the early stage of the reaction, NF was completely covered by the densely packed Ni_3_S_2_ nanoclusters with rough surface, which are formed by the adsorption of active S^2−^ ion on the outer surface of NF leading to the formation of Ni_3_S_2_. The additional Ni^2+^ ions from Ni chloride would result in the growth of Ni-nanosheets on the previously formed Ni_3_S_2_. At this stage, nanosheets were seldom observed, showing that nanosheets have just started to form and deposit on the surface of these densely packed Ni_3_S_2_ nanoclusters (Additional file 1: Figure S2a). It appears that the nucleation centres on the NF trigger the initial growth of densely packed structures. After further hydrothermal reaction, uniform Ni-nanosheets started to deposit resulting in spider web-like structure with exposed edges (Additional file 1: Figure S2b and c). Further extending the reaction time, eventually 10 h onwards, led to the formation of spider web-like nickel nanosheets, completely covering the NF surface and the initially formed Ni_3_S_2_ nanoclusters, with well-defined and exposed edges (Additional file 1: Figure S2d). This phenomenon is quite different to those reported Ni_3_S_2_/NF nanostructures [24, 28, 33–36, 39, 44].

**Fig. 4:**
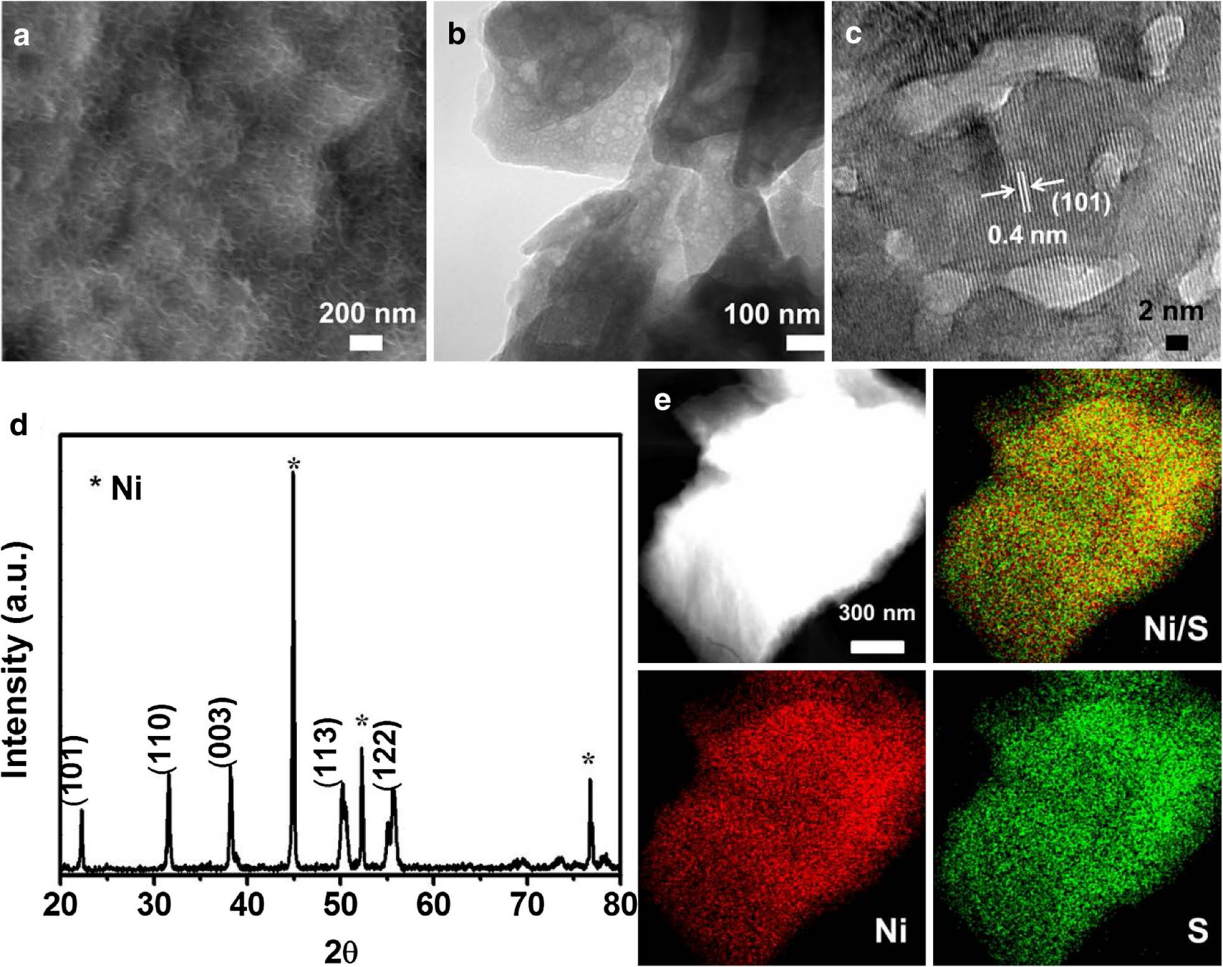
**a** FE-SEM, **b** TEM, **c** HR-TEM images and **d** X-ray diffraction of HC–Ni_3_S_2_/NF. **e** EDS elemental mapping of HC–Ni_3_S_2_ scrapped down from the Ni foam

On the other hand, when the amounts of Ni chloride and thiourea were increased by four-fold and the other experimental conditions were kept same, well-defined honeycomb-like nanosheets were formed as shown in Fig. 4a, b. As discussed above that the nucleation centers on the NF, which trigger the initial growth of the Ni_3_S_2_ structures. However, unlike the formation of spider web-like Ni nanosheets, in the presence of excess amount of Ni and S-precursors, honeycomb-like nanostructure was formed, implying different reaction path. The SEM image (Fig. 4a) demonstrates that the surface of three-dimensional skeleton of NF has been entirely covered by a honeycomb-like porous structure with a large number of exposed holes. Moreover, the HR-TEM image clearly shows that the HC–Ni_3_S_2_ nanostructures are highly crystalline as shown in Fig. 4c and the lattice plane spacing is about 0.40 nm, corresponding to the (101) plane of Ni_3_S_2_ [36]. The XRD pattern in Fig. 4d confirms the presence of nickel sulfide on the NF and all the peaks match well with diffractions of Ni_3_S_2_ (JCPDS card no. 44-1418).

**Fig. 5:**
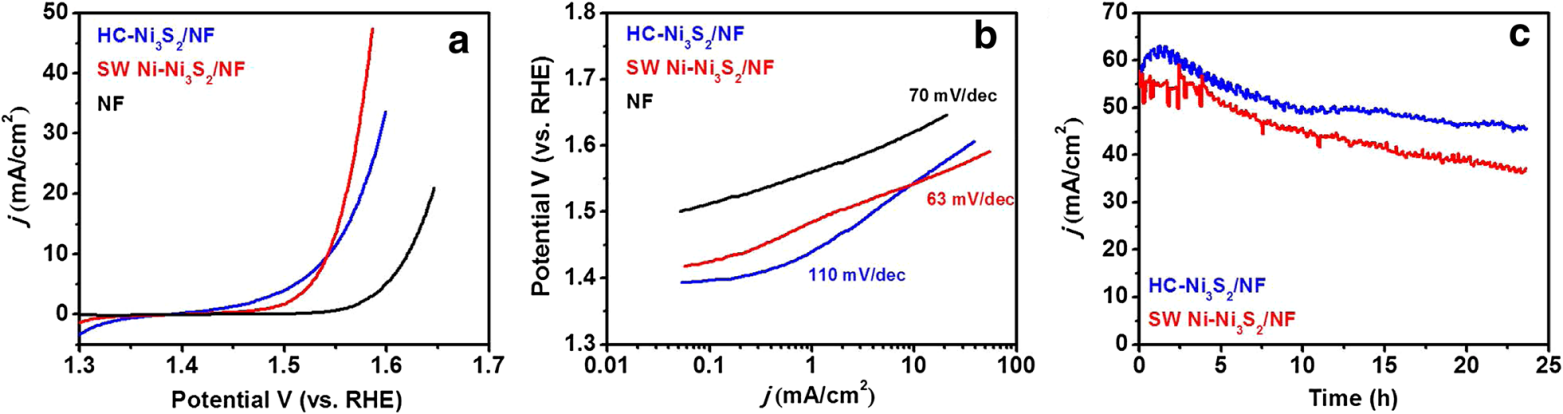
Electrochemical OER performance. **a** Polarization curves of SW Ni–Ni_3_S_2_/NF, HC–Ni_3_S_2_/NF and bare NF at a scan rate of 1 mV/s, **b** corresponding Tafel plots and **c** the chronopotentiometry test [Electrolyte: 1 M KOH and CV scan rate 1 mV/s]

**Fig. 6:**
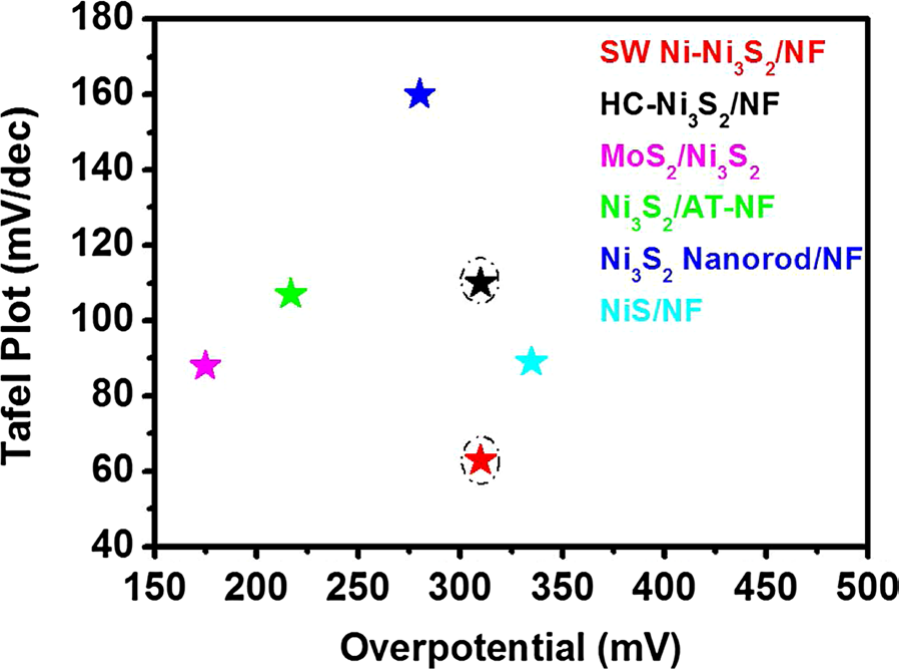
OER activity comparison graph showing Tafel slope (mV/dec) with overpotential (mV) in alkaline media

The performance of electrochemical electrodes is known to depend strongly on the morphology of the electrode materials [38]. In this work, the catalytic activity of as-obtained SW Ni–Ni_3_S_2_/NF and HC–Ni_3_S_2_/NF nanostructures towards oxygen evolution reaction (OER) under alkaline media (pH ~13.5) was evaluated using a typical three-electrode system, in which Ni_3_S_2_/NF nanostructures were directly used as the working electrode (see electrochemical measurement details). Bare NF was also studied for a comparison purpose. Figure 5a shows the linear sweep voltammetry (LSV) of Ni_3_S_2_/NF nanostructures and bare NF within an anodic potential window between 1.3 and 1.7 V vs RHE. Both SW Ni–Ni_3_S_2_/NF and HC–Ni_3_S_2_/NF nanostructures possess earlier onset potential (1.54 V vs RHE) than that of bare NF (1.62 V vs RHE). Furthermore, SW Ni–Ni_3_S_2_/NF shows much larger current density after 1.50 V (vs RHE) than HC–Ni_3_S_2_/NF, indicating that the improvement of electrocatalytic activity for OER. Associated with the favorable morphology and material composition changes, the electrocatalytic activity for OER of the SW Ni–Ni_3_S_2_/NF is markedly enhanced. Therefore, it can be speculated that Ni_3_S_2_ along with the unique spider web-like morphology of Ni-nanosheets with more exposed active sites contributes to the enhancement in the electrocatalytic performance. The electrochemical reactions involved in the OER is inherently surface/interface processes, thus the rate of the reaction is largely depending on the electrochemically active surface area of the electrode [41]. Importantly, the OER activity of these electrocatalysts is observed to be comparable with those of the best reported non-noble metal Ni_3_S_2_/NF based OER catalysts in alkaline media (Details are listed in Additional file 1: Table S1). The Tafel behavior, especially the Tafel slope, is an important kinetic parameter that can reveal changes in the apparent OER mechanism. The rate-determining step (rds) for a specific electrode is normally believed to correspond to its Tafel slope for OER. The corresponding Tafel plots of SW Ni–Ni_3_S_2_/NF, HC–Ni_3_S_2_/NF electrodes and bare NF are shown in Fig. 5b. Although both SW Ni–Ni_3_S_2_/NF and HC–Ni_3_S_2_/NF electrodes possesses similar overpotential (310 mV) regardless of the morphology and the composition, the Tafel slope of SW Ni–Ni_3_S_2_/NF (63 mV/dec) is much smaller than that of HC–Ni_3_S_2_/NF (110 mV/dec), illustrating superior activity of SW Ni–Ni_3_S_2_/NF. These results suggest that the nature of the rate determining step (rds) at both types of Ni_3_S_2_ nanostructures is significantly affected by the morphology and the presence of Ni. As far as we know, this value is the smallest one to date for nickel sulfide based OER catalysts as shown in Fig. 6 and in Additional file 1: Table S1. Furthermore, the OER performance of Ni–Ni_3_S_2_/NF catalyst is one of the best among the non-noble metal OER catalysts in terms of overpotential and Tafel slope.

In light of the above discussion, it seems likely that the kinetic properties of the nickel sulfide nanostructures are quite sensitive to the extent of morphology. Although the physical reason for the change in Tafel slope from 110 to 63 mV/dec is currently uncertain, a possible explanation might lie in increased active surface sites in SW Ni–Ni_3_S_2_/NF due to the presence of spider web-like Ni-nanosheets. Secondly, it could be possible that both materials possess different surface active sites, leading to the increasing inhibition of water and hydroxide ion transfer to the HC–Ni_3_S_2_/NF surface. On the other hand, the exposed active surface sites of the spider web like SW Ni–Ni_3_S_2_/NF could hold considerably more water molecules and thus facilitate easy hydroxide ion discharge, leading to the Tafel slope value decrease. Furthermore, as compared with HC–Ni_3_S_2_/NF, SW Ni–Ni_3_S_2_/NF exhibits smaller charge transfer resistance, implying that higher electron transfer efficiency.

The excellent electrocatalytic activity is further examined by electrochemical double layer capacitances (C_dl_) of the catalysts by performing a cyclic voltammetry (CV) in the potential range where the capacitive current (non-Faradaic response) can be obtained. Additional file 1: Figure S3A and B shows the CV of the catalyst measured at different scan rates of 20, 40, 60 and 100 mV/s. The capacitive current (at the centre of the potential window) then was correlated to the scan rate (Additional file 1: Figure S3C), giving a line with the slope of C_dl_, which is proportional to electrochemical surface area (ECSA). It can be seen from Additional file 1: Figure S3C that the electrochemical double-layer capacitance (EDLC) of SW Ni–Ni_3_S_2_/NF is 3.0 mF/cm^2^, whereas the EDLC of HC–Ni_3_S_2_/NF is only 1.1 mF/cm^2^. The result indicates that the SW Ni–Ni_3_S_2_/NF has much larger ECSA and more amount of active sites, thus shedding light on the improved OER performance. To gain in-depth information on the charge transfer resistance (R_ct_), electrochemical impedance spectroscopy (EIS) measurements were carried out at 1.63 V (η = 400 mV) as shown in Additional file 1: Figure S3D. The Nyquist plot derived from the EIS measurement revealed that both SW Ni–Ni_3_S_2_/NF and HC–Ni_3_S_2_/NF evidently demonstrated a lower resistance of 1.30 and 0.94 Ω, respectively, implying that these material have much better utilization of electrons during the electrochemical process. However, the difference in the charge transfer resistance of both samples is not that significant, suggesting the OER activity of the catalysts appears to be more affected by ECSA than the charge transfer resistance, and therefore resulting in the higher OER activity of SW Ni–Ni_3_S_2_/NF. Therefore, the excellent catalytic performance of the SW Ni–Ni_3_S_2_/NF can be attributed to the fact that the directly grown Ni_3_S_2_ binds strongly onto the NF, which gives an easy electron transport pathway between them. Secondly, the spider web-like Ni-structure provides a large electrochemical active area as compared to honeycomb-like structures. Moreover, due to the intrinsically metallic nature of the Ni_3_S_2_ and the exposed edges of Ni-nanosheets provides most of the catalytically active sites, which can be easily accessible to electrons coming from the electrode [33]. Most importantly, the unique spider web-like Ni-structure exposes a great amount of active surface sites to electrolytes, and therefore minimizes the charge transport limitations and shortens the ion diffusion length. Long term stability is another important criterion for the OER catalysts regarding their practical applications. The electrochemical stability of these catalysts was further tested by polarizing the electrode, using chrono-potentiometric method, at a constant applied potential of 1.7 V as shown in Fig. 5c. It can be seen that the both SW Ni–Ni_3_S_2_/NF and HC–Ni_3_S_2_/NF nanostructures electrode exhibited around 80% of its initial activity retention after 24 h, suggesting good durability for OER in alkaline solution.

## Conclusions

In conclusion, the present work reports a facile one-step hydrothermal synthesis of spider web-like Ni–Ni_3_S_2_/NF and honeycomb-likes Ni_3_S_2_/NF nanostructures on 3D conductive NF. The as-obtained nickel sulfide based electrodes exhibited excellent activity and stability toward oxygen evolution reaction (OER). In particular, spider web-like Ni–Ni_3_S_2_/NF displays a very small Tafel slope value of 63 mV/dec. Important features of the SW Ni–Ni_3_S_2_/NF OER catalyst are believed to have played a key role in diminishing the Tafel slope because (1) morphology and active sites have significant effects on the Tafel slope and (2) highly exposed and accessible catalytic active sites of Ni-nanosheets that enhance the OER activity. To the best of our knowledge, this is the first study on the Ni-nanosheets grown on nickel sulfide based catalyst showing effect of morphology and composition on the Tafel slope value and OER performance. This result seems to open up an exciting possibility to further lower the overpotential and Tafel slope value by fine-tuning the metal sulfide structural features.

## Additional file

**Additional file 1.** Supporting information.
