# Supplementary material for: Morphology controlled synthesis of 2-D Ni–Ni3S2 and Ni3S2 nanostructures on Ni foam towards oxygen evolution reaction
Source: Nano Converg. 2017 Mar 28;4:7. doi: 10.1186/s40580-017-0101-6 (PMC6141903; doi:10.1186/s40580-017-0101-6)
Supplement: Supplementary file 1 — Additional file 1. Supporting information. [file 40580_2017_101_MOESM1_ESM.doc]

**Supplementary Information**

**Morphology controlled synthesis of 2-D Ni-Ni_3_S_2_ and Ni_3_S_2_ nanostructures on Ni foam towards oxygen evolution reaction**

Nitin Kaduba Chaudhari, Aram Oh, Young Jin Sa, Haneul Jin, Hionsuck Baik, Sang Gu Kim, Suk Joong Lee, Sang Hoon Joo and Kwangyeol Lee

Department of Chemistry and Research Institute for Natural Sciences, Korea University, Seoul 02841, Republic of Korea

Center for Molecular Spectroscopy and Dynamics, Institute for Basic Science (IBS), Seoul 02841, Republic of Korea.

Department of Chemistry, Ulsan National Institute of Science and Technology (UNIST), Ulsan 44919, Republic of Korea.

Korea Basic Science Institute (KBSI), Seoul 02841, Republic of Korea.

*Corresponding author E-mail: [kylee1@korea.ac.kr](mailto:kylee1@korea.ac.kr) (KL) and [chungru@korea.ac.kr](mailto:chungru@korea.ac.kr) (NKC)

**Supplementary Figures**

**
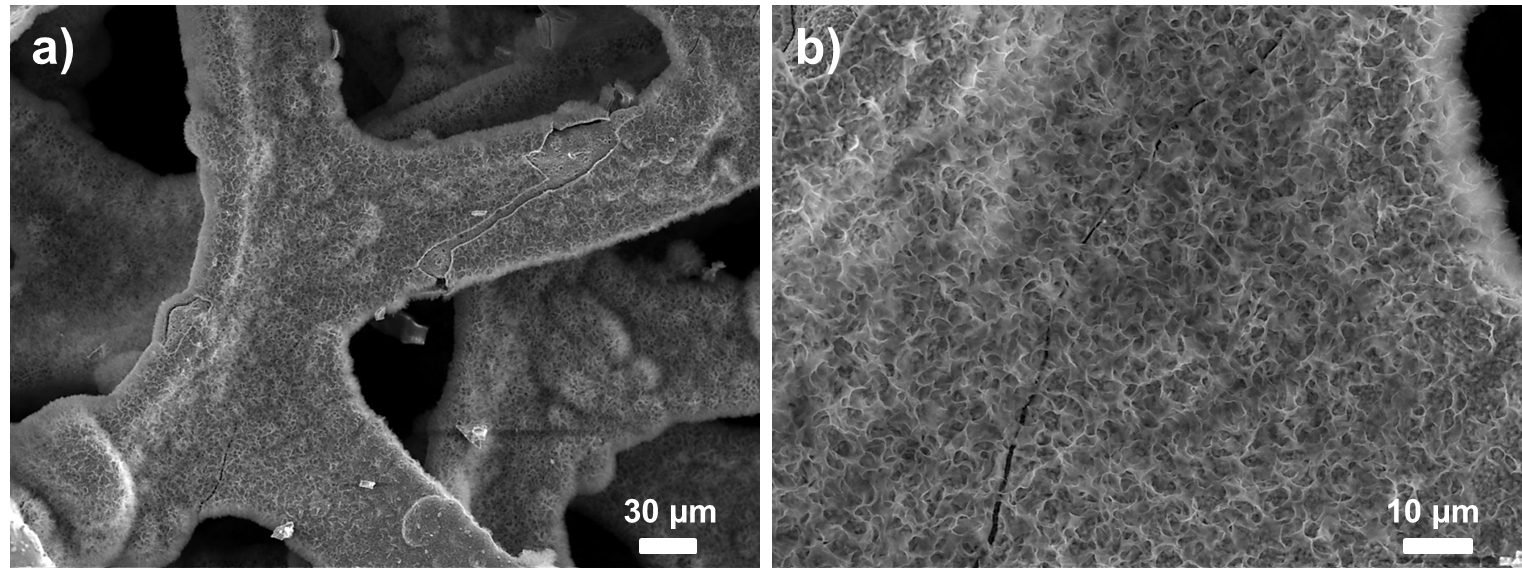
**

**Fig. S1** SEM images of the SW Ni-Ni_3_S_2_/NF at lower magnification.

**
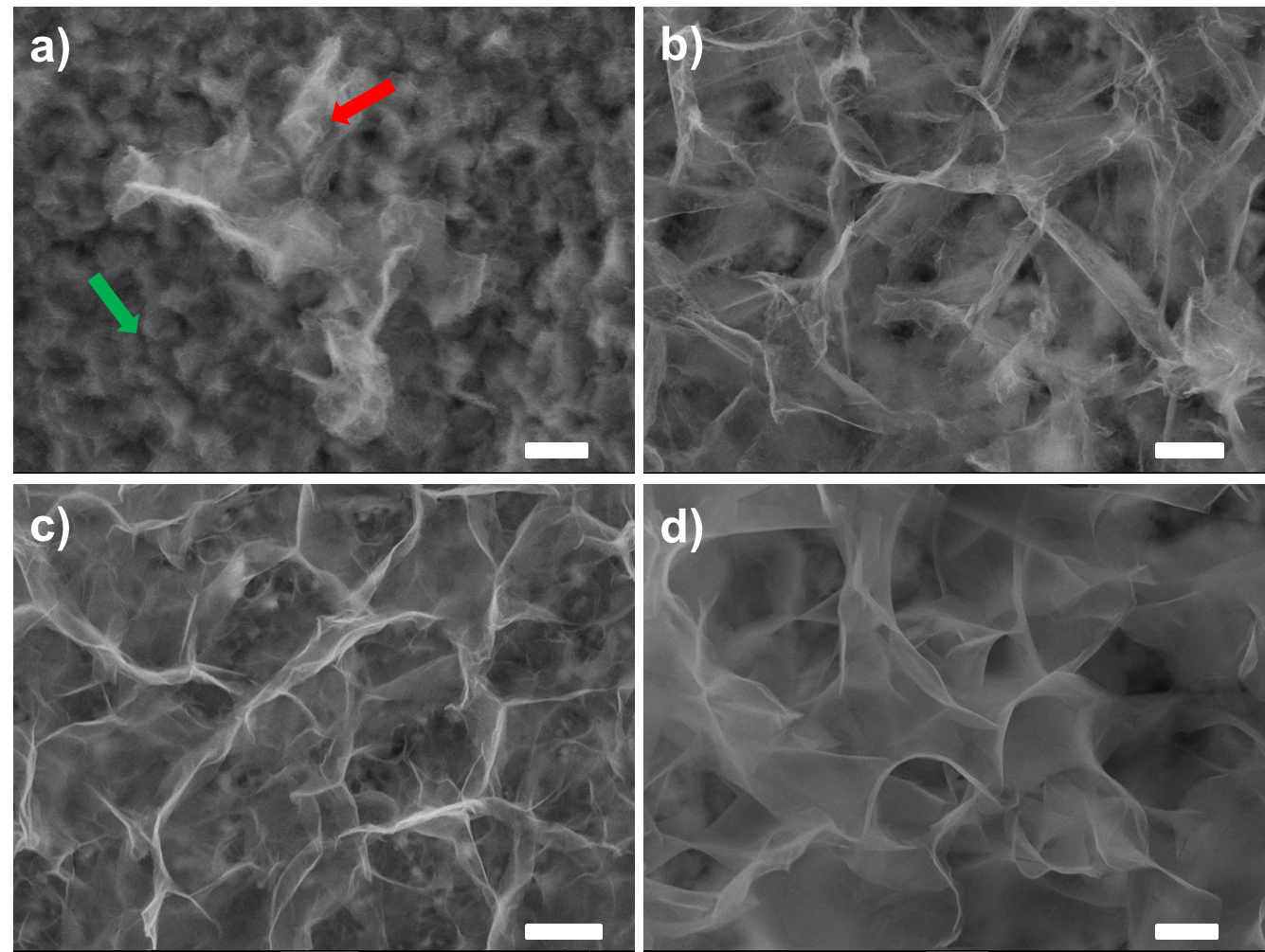
**

**Fig. S2** SEM analysis of the SW Ni-Ni_3_S_2_/NF samples with different reaction durations: a) 1 h, b) 3 h, (c) 7 h, and d) 10 h. Scale bars, 1 µm. The green and red arrows in a) shows the densely packed pillared structures and the initial formation of spider web-like Ni-nanosheets, respectively.

**Table S1: OER activity comparison in** **alkaline media.**

**
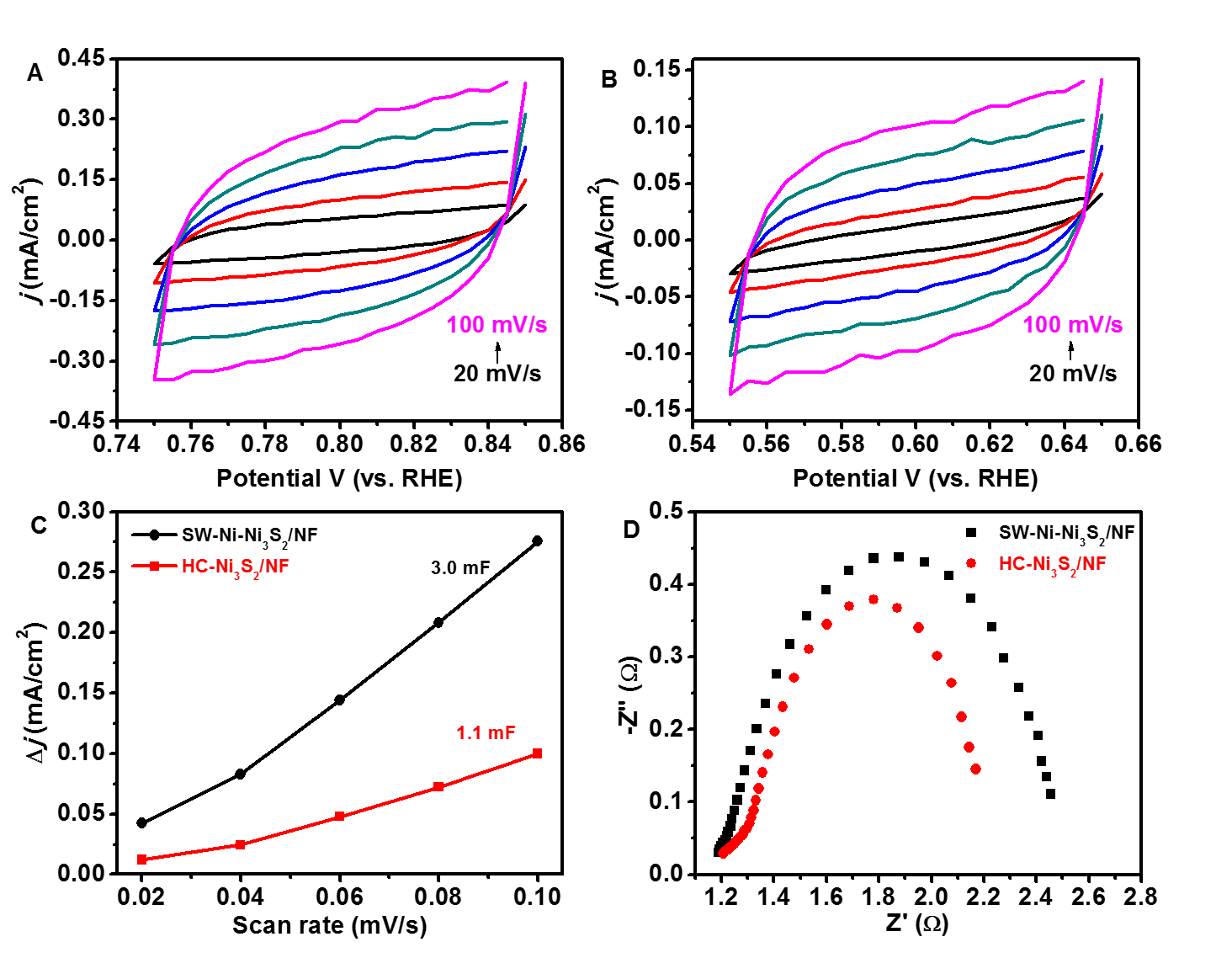
**

**Fig. S3** Electrochemical surface area (ECSA) tests of A) SW Ni-Ni_3_S_2_/NF and B) HC-Ni_3_S_2_/NF catalysts towards OER. C) Charging current density differences plotted against scan rates. D) EIS Nyquist plots at an overpotential of 400 mV.

| **Catalyst** | **Current Density (*j*)**  **(mA/cm^2^)** | **Over-potential at the corresponding *j*** | **Tafel Slope**  **(mV/dec)** | **Ref.** |
| --- | --- | --- | --- | --- |
| SW Ni-Ni_3_S_2_/NF | 10 | 310 | 63 | Present work |
| HC-Ni_3_S_2_/NF | 10 | 310 | 110 | Present work |
| Ni_3_S_2_/AT-NF | 10 | 217 | 107 | 1 |
| Ni_3_S_2_ nanorods/NF | 10 | 280 | 159.3 | 2 |
| Ni_3_S_2_ nanosheets/NF | 10 | 260 | - | 3 |
| NiS/NF | 50 | 335 | 89 | 4 |
| NiFe-LDH/CNT | 10 | 308 | 35 | 5 |
| Mo_3_O_4_/CoSe_2_ | 10 | 450* | 60 | 6 |
| Co-S/Ti mesh | 10 | 361 | 64 | 7 |
| N, S doped Co_x_S_y_@C | 10 | 470 | - | 8 |
| Ni_5_P_4_/Ni foil | 10 | 250 | 40 | 9 |
| NiCo_2_S_4_@N/S-rGO | 10 | 470* | - | 10 |
| NG-CoSe_2_ | 10 | 367 | 40 | 11 |
| NiFe-LDH/NF | 30 | 280 | 50 | 12 |
| NiSe/NF | 20 | 270 | 64 | 13 |
| MoS_2_/Ni_3_S_2_  Heterostructures | 10 | 175 | 88 | 14 |
| Ni_x_S_y_/NF | 10 | 125 | 106 | 15 |

**Note -** * Over-potential value vs Ag/AgCl.

**References for supporting information:**

1. C. Ouyang, X. Wang, C. Wang, X. Zhang, J. Wu, Z. Ma, S. Dou, S. Wang, Electrochimica Acta **74**, 297-301 (2015)
2. W. Zhou, X.J. Wu, X. Cao, X. Huang, C. Tan, J. Tian, H. Liu, J. Wang, H. Zhang, Energy Environ. Sci. **6**, 2921-2924 (2013)
3. L.L. Feng, G. Yu, Y. Wu, G.D. Li, H. Li, Y. Sun, T. Asefa, W. Chen, X. Zou, J. Am. Chem. Soc. **137**, 14023-14026 (2015)
4. W. Zhu, X. Yue, W. Zhang, S. Yu, Y. Zhang, J. Wang, J. Wang, Chem. Commun. **52**, 1486-1489 (2016)
5. M. Gong, Y. Li, H. Wang, Y. Liang, J.Z. Wu, J. Zhou, J. Wang, T. Regier, F. Wei, H. Dai, J. Am. Chem. Soc. **135**, 8452-8455 (2013)
6. M.R. Gao, Y.F. Xu, J. Jiang, Y.R. Zheng, S.H. Yu, J. Am. Chem. Soc. **134**, 2930-2933 (2012)
7. T. Liu, Y. Liang, Q. Liu, X. Sun, Y. He, A. M. Asiri, Electrochem. Commun. **60**, 92-96 (2015)
8. B. Chen, R. Li, G. Ma, X. Gou, Y. Zhu, Y. Xia, Nanoscale **7**, 20674-20684 (2015)
9. M. Ledendecker, S.K. Calderón, C. Papp, H.P. Steinrück, M. Antonietti, M. Shalom, Angew. Chem. **127**, 12538-12542 (2015)
10. Q. Liu, J. Jin, J. Zhang, ACS Appl. Mater. Interfaces **5**, 5002-5008 (2013)
11. M.R. Gao, X. Cao, Q. Gao, Y.F. Xu, Y.R. Zheng, J. Jiang, S.H. Yu, ACS Nano **8**, 3970-3978 (2014)
12. Z. Lu, W. Xu, W. Zhu, Q. Yang, X. Lei, J. Liu, Y. Li, X. Sun, X. Duan, Chem. Commun. **50**, 6479-6482 (2014)
13. C. Tang, N. Cheng, Z. Pu, W. Xing, X. Sun, Angew.Chem. Int .Ed. **54**, 9351-9355 (2015)
14. J. Zhang, T. Wang, D. Pohl, B. Rellinghaus, R. Dong, S. Liu, X. Zhuang, X. Feng, Angew. Chem. Int. Ed. **55**, 6702-6707 (2016)
15. X. Shang, X. Li, W.H. Hu, B. Dong, Y.R. Liu, G.Q. Han, Y.M. Chai, Y.Q. Liu, C.G. Liu, Appl. Surf. Sci. **378**, 15-21 (2016)
